# Up to Four‐Year Outcomes of Single‐Fraction Stereotactic Radiosurgery for Small Brain Metastases: A Competing‐Risk Analysis

**DOI:** 10.1002/cam4.71557

**Published:** 2026-08-06

**Authors:** Isabella Gruber, Oliver Koelbl

**Affiliations:** ^1^ Department of Radiation Oncology University Hospital Regensburg Regensburg Germany

**Keywords:** brain metastases, competing risk, radioresistant cancer types, stereotactic radiosurgery

## Abstract

**Introduction:**

Single‐fraction stereotactic radiosurgery (SRS) is recommended for small brain metastases (< 2 cm). However, long‐term outcomes accounting for competing risks—particularly death, which precludes observation of local treatment failure (LTF)—remain limited.

**Methods:**

We retrospectively analyzed cumulative incidences of LTF, defined as tumor regrowth within the planning target volume, following 18 Gy single‐fraction SRS (July 2014–December 2021) in patients with brain metastases. Patients with prior radiotherapy or surgery to the index lesion were excluded. Cumulative incidences of LTF, death without prior LTF, overall survival (OS), and progression‐free survival (PFS) were estimated. Outcomes were compared between radioresistant (melanoma, sarcoma, renal cell carcinoma) and radiosensitive tumors. Subdistribution hazard ratios (SDHRs) and cause‐specific hazard ratios (CSHRs) identified risk factors for each endpoint.

**Results:**

Seventy‐two patients (median age 62 years, IQR 55–68) with median tumor volume 0.3 cc (IQR 0.2–0.7) were followed for a median of 57.2 months (IQR 49.3–85.4). Thirty‐three tumors were radioresistant and 39 radiosensitive. At 36 months, cumulative LTF was 62% (95% CI, 42%–77%) versus 31% (95% CI, 17%–46%) in radioresistant versus radiosensitive tumors (*p* = 0.011), whereas death without prior LTF was 19% (95% CI, 7%–34%) versus 46% (95% CI, 30%–61%) (*p* = 0.003). OS was 42% (95% CI, 28%–63%) and 36% (95% CI, 24%–55%), and PFS was 19% (95% CI, 9%–40%) and 23% (95% CI, 13%–41%), respectively. Radioresistant histology was associated with higher LTF hazard (CSHR 3.19; SDHR 3.91) and lower hazards of death without prior LTF (CSHR 0.37; SDHR 0.24), with no significant effect on OS or PFS. The 36‐month cumulative incidence of radiation necrosis was 10% (95% CI, 4%–18%).

**Conclusion:**

Local control after 18 Gy SRS is poor in radioresistant brain metastases. Higher LTF incidence is amplified by lower competing mortality, highlighting the interplay between tumor biology and survival. These findings support competing risk–based analyses and consideration of alternative dose‐escalation for high‐risk tumors.

## 1 Introduction

Single‐fraction stereotactic radiosurgery (SRS) is a guideline‐recommended standard of care for the local treatment of brain metastases, strongly endorsed by the American Society for Radiation Oncology (ASTRO) for patients with a limited number of intact metastases < 2 cm in diameter [1]. Prospective trials support single‐fraction SRS as a noninvasive, time‐efficient treatment with a steep dose gradient that spares surrounding brain tissue [2, 3]. Neurosurgical resection is generally reserved for mass effect requiring decompression or the need for histopathological confirmation [1, 4]. While retrospective studies suggest that multi‐fraction stereotactic radiotherapy (FSRT) may improve local control for larger metastases [5, 6], phase III evidence is lacking, making single‐fraction SRS the best‐established modality with strong guideline support [1].

Reported rates of local treatment failure (LTF), defined as tumor regrowth within the planning target volume (PTV), vary widely and depend on lesion size, dose, prescription techniques, and statistical methods. Most studies report short‐term outcomes and use Kaplan–Meier methodology, which censors deceased patients as if they remained at risk for LTF, thereby ignoring death as a competing event and overestimating failure probabilities. This bias is particularly relevant in frail patients with limited life expectancy, in whom premature mortality may equal or exceed the incidence of LTF [7, 8]. Using Kaplan–Meier methods, 1‐year local progression‐free survival (LPFS) for small metastases (~1 cm) ranges from 56% after 18 Gy to 73% after 20 Gy, suggesting a dose–response relationship [6, 9, 10, 11].

In a previous retrospective analysis, we evaluated LPFS following single‐fraction 18 Gy SRS for small brain metastases (~1 cm) with a median follow‐up of 50.8 months (interquartile range [IQR], 30.4–64.6) [12]. To address the limitations of Kaplan–Meier estimates in this frail population, we reanalyzed the cohort using the cumulative incidence function (CIF), which accounts for deaths without prior LTF and provides a realistic estimate of failure in real‐world settings.

The primary objective was to assess the impact of competing mortality on LTF estimation. Secondary objectives were to compare cumulative incidences of LTF between radioresistant histologies (melanoma, sarcoma, renal cell carcinoma) and more radiosensitive tumors, and to evaluate the composite endpoint of PFS and overall survival (OS). Multivariable analyses included Fine–Gray subdistribution hazard ratios and cause‐specific Cox proportional hazards models to evaluate the effects of prognostic factors on LTF, death without prior LTF, PFS, and OS, acknowledging the different interpretations of each model.

## 2 Methods

### 2.1 Patient Selection

We retrospectively analyzed outcomes of patients with brain metastases who received 18 Gy single‐fraction stereotactic radiosurgery (SRS) between September 2014 and December 2021 at the Department of Radiation Oncology, University Hospital Regensburg [12]. Eligible patients had intact brain metastases from solid tumors and received single‐fraction SRS at a prescription dose of 18 Gy without concurrent whole‐brain radiotherapy (WBRT). Only treatment‐naïve brain metastases, defined as lesions that had not received prior local therapy (e.g., stereotactic radiotherapy, whole‐brain radiotherapy, or surgical resection), were included in this analysis. Exclusion criteria were prior WBRT, prior stereotactic radiotherapy (single‐fraction SRS or multifractionated stereotactic radiotherapy, FSRT), or surgical resection of the index lesion. Patients lacking baseline or posttreatment magnetic resonance imaging (MRI) were also excluded.

Of 81 patients who underwent single‐fraction SRS during the study period, seven with prior WBRT and two who died before the first follow‐up MRI were excluded, leaving 72 patients for analysis. To minimize confounding due to multiple brain metastases, the analysis was restricted to the first metastasis treated with 18 Gy single‐fraction SRS in chronological order. Only the first treated lesion per patient was analyzed to meet the independence assumption of competing risk models, as including multiple correlated metastases could bias hazard estimates [13]. The median number of brain metastases treated per patient with 18 Gy SRS was 1.0 (IQR, 1.0–2.0).

### 2.2 Clinical Data

Clinical data were extracted from electronic medical records at the University Hospital Regensburg. All patients were discussed in a multidisciplinary tumor board, and the diagnosis of brain metastases was confirmed by MRI prior to single‐fraction SRS. Collected variables included patient age at SRS, sex, primary diagnosis, UICC stage (8th edition) at cancer diagnosis, date of SRS, gross tumor volume (GTV), planning target volume (PTV), lesion location, primary tumor control, presence of extracranial metastases, and Karnofsky performance status (KPS). Malignant melanoma, renal cell carcinoma, and sarcoma were classified as radioresistant [14, 15]; all other histologies were considered radiosensitive.

Prognostic assessments included the Recursive Partitioning Analysis (RPA) by Gaspar et al. [16] and the updated diagnosis‐specific Graded Prognostic Assessment (ds‐GPA) by Sperduto et al. [17]. Age, KPS, and prognostic scores were assessed on the time of single‐fraction SRS, whereas extracranial disease status and primary tumor control were determined at the most recent evaluation before SRS. Primary tumor control was defined as either surgical resection of the primary tumor or the presence of stable disease (including locoregional lymph nodes) following radiotherapy, as documented at the most recent staging. Systemic therapies with potential CNS activity were also recorded if administered within 3 months before or after SRS. These included molecularly targeted agents (EGFR, ALK, ROS1, and BRAF inhibitors) and immune checkpoint inhibitors (atezolizumab, nivolumab, pembrolizumab).

The outcomes of interest included the cumulative incidence of LTF within the planning target volume (PTV), the competing risk of death without prior LTF, cerebral progression outside the PTV, brain radiation necrosis, PFS, and overall survival (OS).

The study was approved by the Ethics Committee of the University of Regensburg (ethics approval number: 23‐3451‐104, date: July 11, 2023). All patients gave written informed consent.

### 2.3 Single‐Fraction Stereotactic Radiosurgery

As previously reported [12], patients were immobilized using a custom‐made stereotactic mask system crafted from thermoplastic material (Brainlab, Munich, Germany). The planning computed tomography (CT) scans were obtained with a slice thickness of 1 mm. Baseline diagnostic contrast‐enhanced T1‐weighted MRI scans were co‐registered with the CT scans. MRIs used for this purpose were required to be no older than 2 weeks. The GTV was delineated on the contrast‐enhanced T1 MRI sequences, and the planning target volume (PTV) was generated with a 2 mm margin around the GTV. All patients underwent single‐fraction SRS with a prescribed dose of 18 Gy, in accordance with institutional guidelines recommending single‐fraction 18 Gy SRS for smaller brain metastases and multi‐fractionated stereotactic radiotherapy (FSRT) for larger lesions, without a fixed size threshold for selection. The dose prescription was 18 Gy delivered to the enclosing 80% isodose, normalized to a Dmax of 100% (22.5 Gy), or 18 Gy = D98, with a D0.03 cc range of 21.6–22.5 Gy accepted [12]. Minor variations in prescription arose from the transition between treatment planning systems (Oncentra with the collapsed cone algorithm and Monaco with Monte Carlo dose calculation), which technically precluded identical prescriptions. These differences were small and considered clinically negligible.

The biologically effective dose (BED) was calculated as 36 Gy using the linear‐quadratic (LQ) model with an alpha/beta ratio of 12 Gy for brain metastases [18]. Single‐fraction SRS treatments were delivered using coplanar or noncoplanar 6 megavoltage (MV) photon beams from an Elekta SynergyS linear accelerator (Elekta Ltd., Crawley, UK). Daily setup verification and patient repositioning were performed using kV X‐ray and cone beam CT imaging [12]. In cases of LTF following 18 Gy SRS, when a consensus decision was made to proceed with a repeated course of radiotherapy, patients were treated with multi‐fractionated stereotactic radiotherapy (FSRT) using a regimen of 6 fractions of 5 Gy in accordance with institutional guidelines.

### 2.4 Follow‐Up and Tumor Assessment

Patients underwent MRI monitoring 6–8 weeks after single‐fraction SRS and every 3 months thereafter, or earlier if neurological deficits developed. Tumor measurements were performed according to the RANO‐BM criteria [19], with LTF defined as a ≥ 20% increase in the longest diameter of the GTV and an absolute increase of ≥ 5 mm on MRI. Serial MRIs were used to monitor lesion evolution, and LTF and radiation necrosis were adjudicated according to RANO‐BM guidelines, which recognize that post‐SRS enlargement may reflect treatment effect rather than true LTF. In cases where radiographic LTF within the planning target volume (PTV) was suspected but clinical findings suggested brain radiation necrosis rather than true LTF, follow‐up MRIs were performed at shorter intervals. For equivocal cases, additional evidence—such as perfusion MRI, MR spectroscopy, or PET—was used when available. Lesions showing stabilization or shrinkage on subsequent follow‐up were considered indicative of treatment effect and not classified as LTF. If subsequent testing confirmed LTF, the date of LTF was recorded as the date of the scan on which this was first detected [19]. At our university hospital, all patients were presented post‐treatment in a multidisciplinary tumor board with a neuroradiologist in attendance. Persistent uncertainty regarding LTF was resolved by consensus within this board. If subsequent imaging or pathology confirmed treatment response, LTF, or radiation necrosis, the date of the initial scan indicating change was recorded as the event date [19].

Lesions < 1 cm were treated if the interdisciplinary tumor board, including a neuroradiologist, confirmed them as metastases. Lesions < 1 cm deemed not metastases by the neuroradiologist based on imaging characteristics (e.g., contrast enhancement) were observed and not counted as new lesions or included in LTF analyses. The data collection period ended in March 2025.

### 2.5 Definition of Statistical Endpoints

The primary endpoints were cumulative incidence of LTF within the planning target volume (PTV) and death without prior LTF. Death was treated as a competing risk, as it precludes the occurrence of LTF and must be accounted for to avoid overestimating the true probability of LTF. Secondary endpoints included the cumulative incidence of cerebral progression outside the PTV (i.e., new brain metastases), brain radiation necrosis, PFS, and overall survival (OS). All time‐to‐event analyses were calculated from the date of single‐fraction SRS (day 0).

Competing risk methodology was applied using the CIF. For analyses of LTF and cerebral progression, death without the event of interest was treated as a competing event. LTF was defined as recurrence or progression within the initial PTV, characterized by a ≥ 20% increase in the longest diameter of the GTV [19] and an absolute increase of ≥ 5 mm on MRI. Cerebral progression outside the PTV was defined as new brain metastases beyond the treated volume. LTF and death without prior LTF were evaluated using multivariable subdistribution hazard ratios (SDHR) to account for competing events.

Overall survival (OS) was defined as the time from single‐fraction SRS to death from any cause. PFS was defined as the time from SRS to either LTF or death from any cause, representing a composite endpoint that does not differentiate between LTF and death. Patients who remained event‐free were censored at the date of their last documented follow‐up with confirmed event‐free status. Overall survival (OS) and PFS were analyzed using cause‐specific Cox proportional hazards models (CSHR).

Both cause‐specific hazard ratios (CSHR) and subdistribution hazard ratios (SDHR) were estimated to evaluate factors associated with LTF and death without prior LTF. The cause‐specific hazard (CSH) model, implemented via a Cox proportional hazards approach—analogous to Kaplan–Meier estimation—estimates the instantaneous risk of LTF among patients still event‐free, censoring those who die without prior LTF. This approach reflects the biological effect of covariates but may overestimate the cumulative incidence when competing mortality is high. In contrast, the SDHR, estimated using the Fine–Gray model, accounts for competing events such as death without prior LTF and reflects the cumulative incidence of LTF in the real‐world population. Together, these complementary approaches distinguish between covariate effects on the cause‐specific hazard (CSHR) and the observed probability of LTF (SDHR), providing a comprehensive assessment of factors influencing LTF after single‐fraction SRS [8, 20].

### 2.6 Statistical Methods

Patient and treatment characteristics were presented as medians with interquartile ranges (IQR) for continuous variables, and as absolute and relative frequencies for categorical variables. Continuous variables were compared using the Mann–Whitney *U* test, while categorical variables were analyzed using the chi‐square test of independence. Time‐to‐event endpoints (LTF, death without prior LTF) were analyzed using Cox models for cause‐specific hazard ratios (CSHR) and Fine–Gray models for subdistribution hazard ratios (SDHR). Overall survival (OS) and PFS were assessed using Cox proportional hazards regression models. Hazard ratios (HR) and 95% confidence intervals (95% CI) were reported as effect estimates. The proportional hazards assumptions were tested using Schoenfeld‐type residuals. Median follow‐up time was estimated using the reverse Kaplan–Meier method [21], while OS and PFS were estimated using the Kaplan–Meier method. All *p* values were two‐sided, with *p* < 0.05 considered statistically significant. Statistical analyses were performed using R software (version 4.3.2; The R Foundation for Statistical Computing) with the “tidycmprsk,” “survival,” “survminer,” “ggplot2,” “ggsurvfit,” and “gtsummary” packages.

## 3 Results

### 3.1 Patient Characteristics

The median follow‐up was 57.2 months (IQR, 49.3–85.4), and the median patient age at the time of single‐fraction SRS was 62 years (IQR, 55–68). The distribution of cancer types was as follows: malignant melanoma (*n* = 30, 41.7%), NSCLC adenocarcinoma (*n* = 21, 29.2%), NSCLC non‐adenocarcinoma (*n* = 10, 13.9%), breast cancer (*n* = 4, 5.6%), gastrointestinal carcinoma (*n* = 3, 4.2%), sarcoma (*n* = 2, 2.8%), renal cell carcinoma (*n* = 1, 1.4%), and adrenal gland cancer (*n* = 1, 1.4%). Of the cohort, 33 patients had radioresistant tumors (malignant melanoma, renal cell carcinoma, or sarcoma), and 39 had radiosensitive histologies. The median follow‐up was comparable between patients with radioresistant tumors (77.2 months; IQR, 50.8–85.4) and radiosensitive tumors (53.1 months; IQR, 47.4–58.3) (*p* = 0.308).

Several clinical characteristics differed between the groups (Table 1). At initial diagnosis, UICC stage IV disease was less frequent in patients with radioresistant tumors (45.5% vs. 82.1%; *p* = 0.001), suggesting later onset of brain metastases. At the time of single‐fraction SRS, disease control of the primary cancer was more common among patients with radioresistant tumors (72.7% vs. 46.2%; *p* = 0.031), largely reflecting surgical resection of cutaneous malignant melanoma, the predominant tumor type in this cohort. GTVs were smaller in patients with radioresistant tumors (median, 0.3 cc vs. 0.4 cc; *p* = 0.021), while planning target volumes (PTVs) were similar (median, 1.4 cc vs. 1.7 cc; *p* = 0.130). The discrepancy between smaller GTV but comparable PTV in radioresistant tumors reflects radiotherapy planning constraints, as PTV size cannot be reduced below the minimum deliverable field (2 cm^2^).

**TABLE 1:** Patient characteristics (*N* = 72).

Characteristics	Total (*N* = 72)	Radioresistant tumors^a^ (*n* = 33)	Radiosensitive tumors^b^ (*n* = 39)	*p*
Patient age, years				
Median (IQR)	62 (55–68)	63 (52–67)	62 (57–68)	0.490
Mean (SD)	61 (12)	59 (12)	62 (12)	
Sex, *n* (%)				
Male	41 (56.9%)	22 (66.7%)	19 (48.7%)	0.155
Female	31 (43.1%)	11 (33.3%)	20 (51.3%)	
Initial UICC stage^c^, *n* (%)				
I, II, III	25 (34.7%)	18 (54.5%)	7 (17.9%)	**0.001**
IV	47 (65.3%)	15 (45.5%)	32 (82.1%)	
Karnofsky performance score				
Median (IQR)	70 (70–80)	70 (70–80)	80 (70–80)	0.596
Primary cancer control^d^, *n* (%)				
Yes	42 (58.3%)	24 (72.7%)	18 (46.2%)	**0.031**
No	30 (41.7%)	9 (27.3%)	21 (53.8%)	
Extracranial metastases, *n* (%)				
Yes	43 (59.7%)	23 (69.7%)	20 (51.3%)	0.150
No	29 (40.3%)	10 (30.3%)	19 (48.7%)	
Molecularly targeted therapy, immune checkpoint inhibitors^e^, *n* (%)				
Yes	52 (72.2%)	26 (78.8%)	26 (66.7%)	0.299
No	20 (27.8%)	7 (21.2%)	13 (33.3%)	
Recursive partitioning analysis, RPA				
Median (IQR)	2.0 (2.0–3.0)	2.0 (2.0–3.0)	2.0 (2.0–3.0)	0.633
Updated diagnosis‐specific graded prognostic assessment, ds‐GPA				
Median (IQR)	2.0 (1.5–3.0)	2.0 (1.5–3.0)	2.0 (2.0–2.75)	0.634
Location of brain metastases, *n* (%)				
Supratentorial	63 (87.5%)	31 (93.9%)	32 (82.1%)	0.166
Infratentorial	9 (12.5%)	2 (6.1%)	7 (17.9%)	
Gross tumor volume, GTV (cc)				
Median (IQR)	0.3 (0.2–0.7)	0.3 (0.1–0.5)	0.4 (0.3–1.0)	**0.021**
Mean (SD)	0.5 (0.55)	0.4 (0.3)	0.7 (0.6)	
Gross tumor volume, GTV, *n* (%)				
< 0.5 cc (or < 1 cm in diameter)	47 (65.3%)	25 (75.8%)	22 (56.4%)	0.135
≥ 0.5 cc (or ≥ 1 cm in diameter)	25 (34.7%)	8 (24.2%)	17 (43.6%)	
Planning target volume, PTV (cc)				
Median (IQR)	1.6 (1.0–2.4)	1.4 (0.9–2.3)	1.7 (1.2–2.7)	0.130
Mean (SD)	1.9 (1.3)	1.6 (0.9)	2.2 (1.5)	

*Note:* Bold values indicate significance at *p* < 0.05.

Abbreviations: CC, cubic centimeters; IQR, interquartile range; SD, standard deviation.

^a^ *Radioresistant tumors*, malignant melanoma, renal cell, and sarcoma.

^b^ *Radiosensitive tumors*, non‐small cell lung cancer, breast cancer, gastrointestinal carcinoma, and adrenal gland cancer.

^c^ *Initial UICC stage*, at the time of diagnosis of cancer.

^d^ *Control of the primary cancer* was defined as either surgical resection of the primary tumor or the presence of stable disease (including locoregional lymph nodes) following radiotherapy, as confirmed at the most recent staging.

^e^ Administration 3 months before or after SRS. Immune checkpoint inhibitors (*n* = 37), molecularly targeted therapies (BRAF inhibitors, *n* = 5; EGFR inhibitors, *n* = 5; ALK/ROS1 inhibitors, *n* = 3; mTOR inhibitor, *n* = 1; multitarget TKI, *n* = 1).

### 3.2 Time‐To‐Event Analyses for All Endpoints

As of March 26, 2025, 33 patients (45.8%) had experienced LTF within the planning target volume (PTV), 29 (40.3%) had died without LTF, and 10 (13.9%) remained alive without LTF. Table 2 presents cumulative incidence comparisons between patients with radioresistant tumors (malignant melanoma, renal cell carcinoma, sarcoma) and those with radiosensitive tumors. The cumulative incidence of LTF within the PTV was significantly higher in patients with radioresistant tumors (Gray's test, *p* = 0.011), while death prior to LTF was significantly lower (Gray's test, *p* = 0.003). Overall survival (log‐rank test, *p* = 0.30) and progression‐free survival rates (log‐rank test, *p* = 0.80) were similar between the two groups.

**TABLE 2:** Cumulative incidence of local treatment failure (LTF) within the planning target volume (PTV), competing risk of death without prior LTF, overall survival and progression‐free survival at specified time points, stratified by radioresistant versus radiosensitive tumors.

% (95% CI)	Total (*N* = 72)	Radioresistant tumors^a^ (*n* = 33)	Radiosensitive tumors^b^ (*n* = 39)	*p*
LTF within the PTV^c^ (competing death without LTF)				
12 months	28% (18–38)	42% (25–59)	15% (6–29)	0.011 (Gray's test)
24 months	38% (26–49)	49% (31–65)	28% (15–43)	
36 months	45% (33–56)	62% (42–77)	31% (17–46)	
48 months	47% (34–58)	62% (42–77)	34% (19–49)	
Death without prior LTF (competing LTF)				
12 months	14% (7–23)	6% (1–18)	21% (10–34)	0.003 (Gray's test)
24 months	28% (18–39)	9% (2–22)	44% (28–59)	
36 months	34% (23–45)	19% (7–34)	46% (30–61)	
48 months	42% (30–53)	22% (9–38)	59% (40–73)	
Overall survival, OS				
12 months	71% (61–82)	64% (49–82)	77% (65–91)	0.30 (log‐rank)
24 months	46% (35–59)	51% (37–72)	41% (28–60)	
36 months	39% (29–52)	42% (28–63)	36% (24–55)	
48 months	28% (19–41)	35% (22–56)	21% (11–40)	
Progression‐free survival, PFS^d^				
12 months	58% (48–71)	52% (37–72)	64% (51–81)	0.80 (log‐rank)
24 months	34% (25–47)	42% (28–63)	28% (17–47)	
36 months	21% (14–33)	19% (9–40)	23% (13–41)	
48 months	12% (6–23)	16% (7–36)	8% (2–26)	

*Note:* Bold values indicate significance at *p* < 0.05.

^a^ *Radioresistant tumors*, malignant melanoma, renal cell, and sarcoma.

^b^ *Radiosensitive tumors*, non‐small cell lung cancer, breast cancer, gastrointestinal carcinoma, and adrenal gland cancer.

^c^ *Local treatment failure*, LTF, was defined as recurrence or progression within the initial planning target volume (PTV), characterized by a ≥ 20% increase in the longest diameter of the gross tumor volume (GTV) and an absolute increase of ≥ 5 mm on MRI.

^d^ PFS is a composite endpoint, defined as time to local treatment failure or death, without differentiation.

**FIGURE 1:**
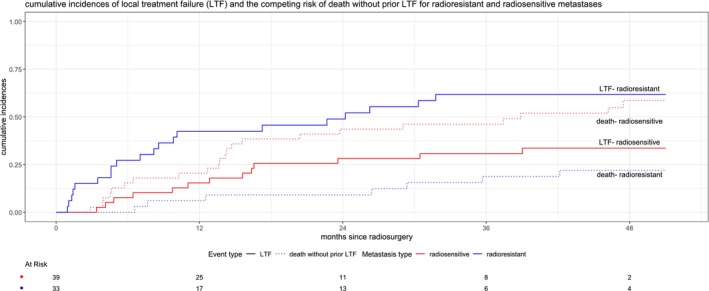
Cumulative incidence of local treatment failure (LTF) of brain metastases within a competing risks framework, accounting for death without prior LTF as a competing event, comparing radioresistant and radiosensitive tumors.

Figure 1 depicts the cumulative incidence of LTF within the planning target volume (PTV) and the competing risk of premature mortality (i.e., death without prior LTF) for radioresistant versus radiosensitive tumors. Brain metastases from radioresistant tumors demonstrated a higher cumulative incidence of LTF but lower competing mortality, whereas metastases from radiosensitive tumors exhibited a lower cumulative incidence of LTF but higher competing mortality.

To assess the impact of tumor size on LTF, metastases were stratified by diameter (< 1 cm vs. ≥ 1 cm), corresponding to a GTV of < 0.5 cc versus ≥ 0.5 cc.

The cumulative incidences of LTF were similar between brain metastases with a GTV ≥ 0.5 cc and those < 0.5 cc (Gray's test, *p* = 0.7). For metastases with a GTV ≥ 0.5 cc, the cumulative incidences of LTF were 24% (95% CI, 9%–42%), 37% (95% CI, 18%–56%), 41% (95% CI, 21%–60%), and 41% (95% CI, 21%–60%) at 12, 24, 36, and 48 months, respectively. For metastases with a GTV < 0.5 cc, the corresponding cumulative incidences of LTF were 30% (95% CI, 17%–43%), 38% (95% CI, 24%–52%), 47% (95% CI, 32%–60%), and 49% (95% CI, 34%–62%), respectively. The cumulative incidence of death without prior LTF was similar between groups (GTV ≥ 0.5 cc vs. < 0.5 cc), with 8.0% (95% CI, 1%–23%), 32% (95% CI, 15%–51%), 41% (95% CI, 21%–60%), and 59% (95% CI, 32%–78%) for GTV ≥ 0.5 cc, and 17% (95% CI, 8%–29%), 26% (95% CI, 14%–39%), 30% (95% CI, 17%–43%), and 35% (95% CI, 21%–49%) for GTV < 0.5 cc at 12, 24, 36, and 48 months, respectively (Gray's test, *p* = 0.12).

Overall survival (OS) rates were similar between patients with GTV ≥ 0.5 cc and those with GTV < 0.5 cc (log‐rank test, *p* = 0.20). OS for GTV ≥ 0.5 cc was 80% (95% CI, 66%–97%), 43% (95% CI, 28%–68%), 30% (95% CI, 17%–56%), and 20% (95% CI, 9%–46%) at 12, 24, 36, and 48 months, respectively, compared with 66% (95% CI, 54%–81%), 47% (95% CI, 35%–64%), 43% (95% CI, 31%–59%), and 31% (95% CI, 20%–48%) for GTV < 0.5 cc.

PFS rates were comparable between GTV ≥ 0.5 cc and GTV < 0.5 cc (log‐rank test, *p* = 0.4). PFS rates at 12, 24, 36, and 48 months were 68% (95% CI, 52%–89%), 31% (95% CI, 17%–56%), 18% (95% CI, 7%–42%), and 0% for GTV ≥ 0.5 cc, and 53% (95% CI, 41%–70%), 36% (95% CI, 25%–53%), 23% (95% CI, 14%–39%), and 16% (95% CI, 8%–32%) for GTV < 0.5 cc, respectively.

Among cases of LTF within the PTV (*n* = 33), patients received FSRT with 6 fractions of 5 Gy (*n* = 14, 42.4%), whole‐brain radiotherapy due to concomitant progression outside the PTV (*n* = 8, 24.2%), surgery (*n* = 6, 18.2%), CNS‐active systemic therapies alone (*n* = 3, 9.1%), or best supportive care (*n* = 2, 6.1%).

### 3.3 Univariate and Multivariate Analyses of Clinical Outcomes

Table 3 summarizes the univariable and multivariable analyses of clinical outcomes using CSH and subdistribution hazard (SDH) models. Radioresistant tumors were associated with a significantly higher risk of LTF within the PTV. The CSHR for LTF was 3.19 (95% CI, 1.44–7.09; *p* = 0.004), indicating a threefold higher instantaneous risk, while the SDHR was 3.91 (95% CI, 1.75–8.76; *p* < 0.001), reflecting a substantially greater cumulative incidence of LTF over time compared with radiosensitive tumors. Conversely, radioresistant tumors were associated with significantly lower competing mortality (death without prior LTF), with an SDHR of 0.24 (95% CI, 0.10–0.58; *p* = 0.002) and a CSHR of 0.37 (95% CI, 0.14–0.93; *p* = 0.035). As a result, despite the increased LTF, radioresistant tumors were not associated with significantly shorter PFS, likely reflecting the counterbalancing effect of lower competing mortality on this composite endpoint.

**TABLE 3:** Univariable and multivariable analysis of clinical outcomes.

	Local treatment failure, LTF^a^ (competing death)		Death without prior LTF^a^ (competing LTF)		Local treatment failure, LTF^a^		Death without prior LTF^a^		Overall survival		Progression‐free survival^b^	
	Fine‐Gray‐analysis		Fine‐Gray‐analysis		Cox regression analysis		Cox regression analysis		Cox regression analysis		Cox regression analysis	
	Subdistribution hazard ratio, SDHR (95% CI)	*p*	Subdistribution hazard ratio, SDHR (95% CI)	*p*	Cause‐specific hazard ratio, CSHR (95% CI)	*p*	Cause‐specific hazard ratio, CSHR (95% CI)	*p*	Cause‐specific hazard ratio, CSHR (95% CI)	*p*	Cause‐specific hazard ratio, CSHR (95% CI)	*p*
Univariable model												
Tumor types												
Radiosensitive (reference)^c^												
Radioresistant^d^	2.43 (1.23–4.80)	**0.011**	0.29 (0.12–0.65)	**0.003**	1.85 (0.92–3.72)	0.085	0.38 (0.16–0.89)	**0.026**	0.76 (0.44–1.30)	0.3	0.93 (0.56–1.53)	0.8
Multivariable model												
Tumor types												
Radiosensitive (reference)^c^												
Radioresistant^d^	3.91 (1.75–8.76)	**< 0.001**	0.24 (0.10–0.58)	**0.002**	3.19 (1.44–7.09)	**0.004**	0.37 (0.14–0.93)	**0.035**	0.89 (0.49–1.62)	0.7	1.13 (0.65–1.97)	0.7
Gross tumor volume, GTV (cc)	1.57 (0.86–2.86)	0.14	0.81 (0.41–1.59)	0.54	1.54 (0.85–2.79)	0.16	1.10 (0.58–2.09)	0.77	1.14 (0.72–1.81)	0.6	1.25 (0.78–1.98)	0.4
Patient age	0.99 (0.96–1.01)	0.26	1.03 (0.98–1.07)	0.25	0.99 (0.97–1.02)	0.48	1.02 (0.98–1.07)	0.27	1.02 (0.99–1.05)	0.15	1.01 (0.99–1.03)	0.5
KPS	1.01 (0.98–1.05)	0.56	0.97 (0.93–1.00)	0.076	0.99 (0.95–1.02)	0.48	0.96 (0.93–1.00)	**0.038**	0.95 (0.93–0.98)	**< 0.001**	0.97 (0.94–0.99)	**0.017**
ICI, TT therapy^e^												
No (reference)												
Yes	0.32 (0.13–0.77)	**0.011**	2.32 (0.92–5.84)	0.075	0.34 (0.15–0.77)	**0.010**	1.54 (0.59–4.06)	0.38	0.83 (0.46–1.50)	0.5	0.65 (0.36–1.18)	0.2

*Note:* Bold values indicate significance at *p* < 0.05.

^a^ *Local treatment failure, LTF* was defined as recurrence or progression within the initial planning target volume (PTV), characterized by a ≥ 20% increase in the longest diameter of the gross tumor volume (GTV) and an absolute increase of ≥ 5 mm on MRI.

^b^ PFS is a composite endpoint, defined as time to local treatment failure or death, without differentiation.

^c^ *Radiosensitive tumors*, non‐small cell lung cancer, breast cancer, gastrointestinal carcinoma, and adrenal gland cancer.

^d^ *Radioresistant tumors*, malignant melanoma, renal cell, and sarcoma.

^e^ *ICI, TT therapy*, Immune checkpoint inhibitors, and molecularly targeted therapies with administration 3 months before or after SRS.

Higher KPS was associated with reduced competing mortality (death without prior LTF) (CSHR 0.96, 95% CI, 0.93–1.00; *p =* 0.038), improved overall survival (CSHR 0.95, 95% CI, 0.93–0.98; *p* < 0.001), and longer PFS (CSHR 0.97, 95% CI, 0.94–0.99; *p* = 0.017). Administration of CNS‐active systemic therapy significantly lowered both the cumulative incidence of LTF (SDHR 0.32, 95% CI, 0.13–0.77; *p* = 0.011) and instantaneous risk of LTF (CSHR 0.34, 95% CI, 0.15–0.77; *p* = 0.010), without a corresponding effect on PFS or overall survival (OS).

### 3.4 Distant Brain Failure

Distant brain failure, defined as cerebral progression outside the PTV, was common. Overall, 47 patients (65.3%) experienced distant brain failure during the course of their disease, 10 (13.9%) remained alive without distant brain failure, and 15 (20.8%) died without developing it. The cumulative incidence of distant brain failure was similar between patients with radioresistant and radiosensitive tumors (Gray's test, *p* = 0.3). Among patients with radioresistant tumors, the cumulative incidences were 43% (95% CI, 25%–59%) at 12 months, 46% (95% CI, 28%–62%) at 24 months, 68% (95% CI, 47%–82%) at 36 months, and 72% (95% CI, 50%–85%) at 48 months. For radiosensitive tumors, corresponding cumulative incidences were 31% (95% CI, 17%–46%), 41% (95% CI, 26%–56%), 48% (95% CI, 31%–63%), and 58% (95% CI, 39%–73%) at 12, 24, 36, and 48 months, respectively.

### 3.5 Brain Radiation Necrosis

Brain radiation necrosis occurred in eight patients (11.1%) following 18 Gy SRS. Seven cases were pathologically confirmed after surgical intervention, while one symptomatic patient exhibited radiographic features consistent with radiation necrosis and improved clinically and radiographically following bevacizumab therapy. All affected patients were receiving systemic therapy at the time of diagnosis, including osimertinib for NSCLC (*n* = 1), pembrolizumab for NSCLC (*n* = 2), lorlatinib for breast cancer (*n* = 1), cytotoxic chemotherapy for gastrointestinal cancer (*n* = 1), and nivolumab for malignant melanoma (*n* = 3). By contrast, 51 patients (70.8%) died before developing radiation necrosis (competing event), and 13 patients (18.1%) remained alive without signs of necrosis at last follow‐up.

**FIGURE 2:**
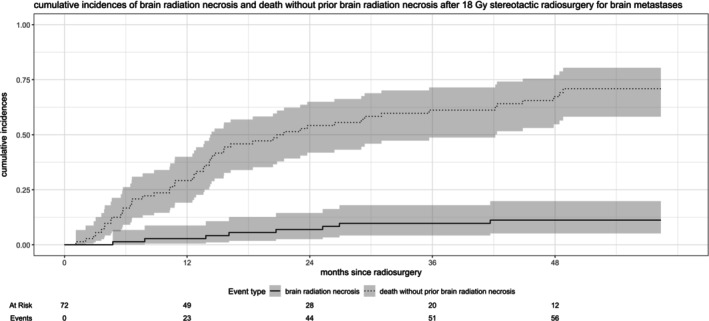
Cumulative incidence of brain radiation necrosis and the competing risk of death without prior brain radiation necrosis following 18 Gy stereotactic radiosurgery for brain metastases, with 95% confidence intervals.

Figure 2 shows the cumulative incidence of brain radiation necrosis and the competing risk of death without prior necrosis. The cumulative incidence of brain radiation necrosis was 3% (95% CI, 1%–9%), 7% (95% CI, 2%–14%), 10% (95% CI, 4%–18%), and 11% (95% CI, 5%–20%) at 12, 24, 36, and 48 months, respectively. Corresponding cumulative incidences for the competing event—death without prior brain radiation necrosis—were 29% (95% CI, 19%–40%), 54% (95% CI, 42%–65%), 61% (95% CI, 49%–71%), and 67% (95% CI, 55%–77%) at 12, 24, 36, and 48 months, respectively.

The cumulative incidence of brain radiation necrosis did not differ significantly between metastases with a GTV < 0.5 cc and those ≥ 0.5 cc (Gray's test, *p* = 0.30). For lesions < 0.5 cc, cumulative incidences at 12, 24, 36, and 48 months were 0%, 4% (95% CI, 1%–13%), 8% (95% CI, 3%–19%), and 8% (95% CI, 3%–19%), respectively. Corresponding incidences for lesions ≥ 0.5 cc were 8% (95% CI, 1%–23%), 12% (95% CI, 3%–28%), 12% (95% CI, 3%–28%), and 17% (95% CI, 5%–36%). The cumulative incidences of the competing event (death without prior brain radiation necrosis) were similar between groups (data not shown, Gray's test, *p* = 0.7).

There were no significant differences in the cumulative incidences of brain radiation necrosis (Gray's test, *p* = 0.6) and the competing event (data not shown, Gray's test, *p* = 0.7) between patients with radioresistant tumors and those with radiosensitive tumors. Cumulative incidences of brain radiation necrosis were 3% (95% CI, 0%–14%), 6% (95% CI, 1%–18%), 9% (95% CI, 2%–22%), and 9% (95% CI, 2%–22%) for radioresistant tumors, and 3% (95% CI, 0%–12%), 8% (95% CI, 2%–19%), 10% (95% CI, 3%–22%), and 13% (95% CI, 5%–26%) for radiosensitive tumors at 12, 24, 36, and 48 months, respectively. CNS‐active immuno‐ or targeted therapies did not affect the cumulative incidence of brain radiation necrosis (data not shown; Gray's test, *p* = 0.3) or the competing event (data not shown; Gray's test, *p* = 0.12).

## 4 Discussion

This retrospective study evaluated outcomes in patients with small brain metastases (~1 cm) treated with a uniform single‐fraction SRS dose of 18 Gy. With a median follow‐up of 5 years, it provides a comprehensive assessment of the long‐term results following single‐fraction SRS. An explicit goal of this manuscript is to raise awareness of the conceptual and practical differences between Kaplan–Meier estimates and CIF when analyzing LTF after radiotherapy in a frail population at high risk of early mortality, for example due to comorbidities. We believe that this distinction is highly relevant for the interpretation of published radiotherapy outcomes and is currently underappreciated in the literature. A central novel finding is that the increased cumulative incidence of LTF in radioresistant tumors is driven not only by higher biological failure risk but also by lower competing mortality, which further amplifies observed failure rates over time. This interaction between tumor biology and competing risks was not addressed in our prior work. Radioresistant tumors showed substantially higher LTF, but PFS was not shorter in this subgroup, likely because fewer deaths before LTF mitigated its impact on the composite endpoint.

In our cohort, 65% of brain metastases were < 1 cm in diameter, and our institution used a standardized single‐fraction SRS dose of 18 Gy. While RTOG 90–05 allowed doses up to 24 Gy for very small lesions, these recommendations were based on maximum tolerated doses rather than proven superiority in local control [3]. Although some centers prescribe 20 Gy for small lesions, there is no prospective evidence demonstrating a clinically meaningful improvement in outcomes compared with 18 Gy, particularly as normalization and handling of competing risks differ substantially. Using a modern prescription of 18 Gy = D98 with accepted hotspot doses of 21.6–22.5 Gy, the effective tumor core dose is comparable to higher nominal prescriptions, suggesting adequate coverage while balancing the risk of toxicity (e.g., brain radiation necrosis). Some retrospective series have suggested that larger single‐fraction doses and higher target coverage may improve radiographic response in metastases from historically radioresistant primaries such as melanoma or renal cell carcinoma; however, other analyses have not demonstrated a consistent effect of histology on local control, and no prospective dose‐escalation data exist specifically for radioresistant histologies [22, 23, 24]. Although the optimal fractionation regimen remains unclear, radiobiological modeling [25] and clinical evidence [7] suggest that multi‐fractionated stereotactic radiotherapy (FSRT) may provide superior local control compared with single‐fraction SRS. FSRT was initially applied to larger metastases (> 3 cm) to reduce toxicity. Retrospective studies indicate that FSRT can improve local control while lowering brain radiation necrosis [5, 11]. However, FSRT has not been directly compared with single‐fraction SRS in a randomized prospective trial, and single‐fraction SRS currently carries a higher level of evidence [1]. The prospective, multicenter FSRT Trial, currently recruiting, will evaluate whether FSRT (12 × 4 Gy) is superior to single‐fraction SRS for local control and radiation necrosis in metastases measuring 1–4 cm.

The findings of the present study only partially align with previous reports, many of which predominantly included patients with non‐small cell lung cancer (NSCLC)—a histology known for its radiosensitivity [26]—or relied on Kaplan–Meier methods rather than the CIF. Kaplan–Meier analysis is known to overestimate relative risk by a median of 6%–10% compared with CIF, particularly when competing events (deaths without prior LTF) occur at similar or higher rates than the primary event (LTF) or when event rates exceed 10% [7, 8]. In the present cohort, 40.3% of patients died before developing LTF of irradiated brain metastases, mostly due to extracranial disease progression and comorbidities, rendering these deaths significant competing events [7, 8]. In their review of 100 published studies, van Walraven and McAlister found that 46% did not properly account for competing risks, leading to a median relative risk overestimation of 6% using Kaplan–Meier methods, with more than one‐third of studies overestimating by over 10% [7]. These methodological limitations can bias clinical interpretation, particularly in elderly or metastatic populations, where early mortality is common [27].

The reduced tumor control observed in melanoma aligns with previously reported data [9, 28]. A recent study using Kaplan–Meier analysis reported a 1‐year local control rate of 55.6% following a median single‐fraction SRS dose of 18 Gy for brain metastases measuring 1 cm in diameter [9]. In that study, 55% of patients had malignant melanoma—a proportion of radioresistant tumors similar to the 46% observed in the present study.

Tumor size is well established as a predictor of LTF after SRS, with larger brain metastases showing higher rates of LTF. In this analysis, GTV was evaluated as a continuous variable in multivariate analyses to avoid information loss associated with arbitrary dichotomization. Nevertheless, GTV was not significantly associated with LTF or brain radiation necrosis. This may be due to the limited number of brain metastases and events, the impact of competing risks such as early death, and institutional treatment selection, as brain metastases treated with single‐fraction 18 Gy SRS were typically very small and not considered for multifractionated regimens. Consequently, the restricted variability in tumor size may have attenuated the detectable effect of GTV, suggesting that the lack of association reflects methodological and cohort‐related factors rather than a true absence of a size effect.

New brain metastases outside the planning target volume (PTV) remain a major limitation of stereotactic radiotherapy. Although SRS alone is associated with higher rates of intracranial progression than combined whole‐brain radiotherapy (WBRT) and SRS, omission of upfront WBRT does not appear to compromise overall survival [2, 29], likely reflecting the effectiveness of salvage strategies. In our cohort, most patients with LTF (42.4%) received salvage multi‐fractionated stereotactic radiotherapy (6 × 5 Gy).

Although combining single‐fraction 18 Gy SRS with CNS‐active targeted agents or immune checkpoint inhibitors was associated with lower LTF [30], this did not translate into a survival benefit in our cohort. The cumulative incidence of brain radiation necrosis in this study was 10% at 36 months, with pathological confirmation in all but one patient. However, the limited number of events precluded multivariable analysis. Comparisons with the literature are challenging due to differences in statistical methods, fractionation schemes, and metastasis size. Retrospective data for small metastases treated with 18 Gy SRS report radionecrosis rates of 14.8% at 12 months using Kaplan–Meier methods, which likely overestimate incidence and do not capture longer‐term outcomes [7]. Minniti et al. reported a 1‐year cumulative incidence of 18% following 18 Gy single‐fraction SRS for 2–3 cm lesions, remaining below ~30% at 24 months, while multi‐fraction SRS reduced 1‐year rates to 9% and subsequent rates below 20% [5].

The literature shows considerable interinstitutional variability in single‐fraction SRS, including differences in prescription patterns, doses, and statistical approaches, with some studies using Kaplan–Meier estimates and others employing competing risk analysis via the CIF. Such heterogeneity complicates direct comparisons between studies and underscores the need for standardized treatment protocols and analytic approaches to enable more consistent and reliable outcome reporting across institutions.

This study has several limitations, including its retrospective design and relatively small sample size, which limit the generalizability of the findings. The lack of a clear association between GTV and LTF observed in this analysis is likely due to limited statistical power, the presence of competing events, and potential treatment selection effects, rather than a true absence of effect. The cohort included an unusually high proportion of melanoma metastases (42%), which may limit generalizability to populations with more typical distributions of primary tumors. The predominance of radioresistant tumors in our sample may also influence the observed LTF and the effect of competing risks, and should be considered when interpreting the results. A key limitation is the lack of histological confirmation of LTF, as surgical resection was performed in only a small subset. LTF was primarily assessed by MRI, with amino acid PET‐CT used when findings were inconclusive. Distinguishing true LTF from radiation necrosis is inherently challenging, as postradiation changes can mimic tumor growth within or adjacent to the PTV. Follow‐up imaging and multidisciplinary tumor board review were used to enhance diagnostic accuracy and differentiate true LTF from radiation necrosis.

CNS‐active systemic therapy was recorded as a categorical variable based on administration within 3 months before or after SRS. The exact initiation timing and therapies outside this window were not captured, so the reported impact may not fully reflect the true effect. Incorporating time‐varying covariates into the subdistribution hazard model would have precluded reliable evaluation of covariate effects on the CIF in the presence of competing risks [20]. Thus, the applied model is considered statistically appropriate for this analysis. Finally, while we analyzed only the first treated lesion per patient to satisfy independence assumptions, this approach may preferentially include earlier‐detected lesions.

## 5 Conclusion

Patients with radioresistant brain metastases (melanoma, sarcoma, renal cell carcinoma) showed a markedly higher cumulative incidence of LTF, reflecting both intrinsic tumor resistance and a lower incidence of death prior to LTF. Despite higher LTF, PFS was not shorter in this subgroup, likely because fewer deaths before LTF reduced its impact on the composite endpoint. Single‐fraction 18 Gy SRS was generally well tolerated, although the optimal fractionation for radioresistant tumors remains undefined. Future studies should explore dose optimization, alternative fractionation strategies, and integration of CNS‐active systemic therapies to improve intracranial control.

Our analysis also highlights the value of competing risk methodology using the CIF. In the CSH model—analogous to Kaplan–Meier—patients who die before LTF are treated as censored, so only those still at risk contribute to the hazard; the CSHR reflects the biological effect of covariates on LTF but not the observed probability of failure. In contrast, the Fine–Gray subdistribution hazard (SDHR) model retains deaths without prior LTF in the risk set, weighted appropriately, providing a realistic estimate of cumulative incidence. SDHR thus reflects the observed cumulative incidence of LTF, whereas CSHR reflects intrinsic biological effects. Importantly, in the absence of competing events, Kaplan–Meier and CIF yield identical cumulative incidence estimates, highlighting that differences only arise when competing mortality is present. Therefore, standardized CIF‐based reporting should be preferred for accurate interpretation of treatment efficacy and comparability across studies, particularly in populations with limited life expectancy.

## Author Contributions

**Isabella Gruber:** methodology; project administration; conceptualization; validation; data curation; formal analysis; writing – original draft; writing – review and editing. **Oliver Koelbl:** writing – review and editing; resources.

## Funding

The authors have nothing to report.

## Ethics Statement

This retrospective study was approved by the local Ethics Board of the University of Regensburg (approval number 23–3451‐104).

## Consent

All patients gave written informed consent.

## Conflicts of Interest

The authors declare no conflicts of interest.

## Data Availability

The data that support the findings of this study are available on request from the corresponding author. The data are not publicly available due to privacy or ethical restrictions.
